# Exogenous Cripto-1 Suppresses Self-Renewal of Cancer Stem Cell Model

**DOI:** 10.3390/ijms19113345

**Published:** 2018-10-26

**Authors:** Md Jahangir Alam, Ryota Takahashi, Said M. Afify, Aung Ko Ko Oo, Kazuki Kumon, Hend M. Nawara, Aprilliana Cahya Khayrani, Juan Du, Maram H. Zahra, Akimasa Seno, David S. Salomon, Masaharu Seno

**Affiliations:** 1 Department of Medical Bioengineering, Graduate School of Natural Science and Technology, Okayama University, Okayama 700-8530, Japan; pw206tfp@s.okayama-u.ac.jp (M.J.A.); pv3510pn@s.okayama-u.ac.jp (R.T.); saidafify@s.okayama-u.ac.jp (S.M.A.); ps5g1bh2@s.okayama-u.ac.jp (A.K.K.O.); en419781@s.okayama-u.ac.jp (K.K.); pnt32hvi@s.okayama-u.ac.jp (H.M.N.); phrw1rm8@s.okayama-u.ac.jp (A.C.K.); pirh7vbs@s.okayama-u.ac.jp (J.D.); 2 Department of Genetic Engineering and Biotechnology, Shahjalal University of Science and Technology, Sylhet 3114, Bangladesh; jahangir-geb@sust.edu; 3 Division of Biochemistry, Chemistry Department, Faculty of Science, Menoufia University, Shebin ElKoum-Menoufia 32511, Egypt; 4 Laboratory of Nano-Biotechnology, Graduate School of Interdisciplinary Science and Engineering in Health Systems, Okayama University, Okayama 700-8530, Japan; maram@okayama-u.ac.jp (M.H.Z.); aseno@wayne.edu (A.S.); 5 Okayama University Research Laboratory of Stem Cell Engineering in Detroit, IBio, Wayne State University, Detroit, MI 48202, USA; 6 Mouse Cancer Genetics Program, Center for Cancer Research, National Cancer Institute, Frederick, MD 21702, USA; salomond@mail.nih.gov

**Keywords:** mouse iPS, miPS-LLCcm, Cripto-1, recombinant Cripto-1, self-renewal, cancer stem cells

## Abstract

Cripto-1 is a glycophosphatidylinositol (GPI) anchored signaling protein of epidermal growth factor (EGF)-Cripto-1-FRL1-Cryptic (CFC) family and plays a significant role in the early developmental stages and in the different types of cancer cells, epithelial to mesenchymal transition and tumor angiogenesis. Previously, we have developed cancer stem cells (miPS-LLCcm) from mouse iPSCs by culturing them in the presence of conditioned medium of Lewis Lung Carcinoma (LLC) cells for four weeks. Nodal and Cripto-1 were confirmed to be expressed in miPS-LLCcm cells by quantitative reverse transcription PCR (rt-qPCR) implying that Cr-1 was required in maintaining stemness. To investigate the biological effect of adding exogenous soluble CR-1 to the cancer stem cells, we have prepared a C-terminally truncated soluble form of recombinant human CR-1 protein (rhsfCR-1), in which the GPI anchored moiety was removed by substitution of a stop codon through site-directed mutagenesis. rhsfCR-1 effectively suppressed the proliferation and sphere forming ability of miPS-LLCcm cells in a dose-dependent manner in the range of 0 to 5 µg/mL, due to the suppression of Nodal-Cripto-1/ALK4/Smad2 signaling pathway. Frequency of sphere-forming cells was dropped from 1/40 to 1/69 by rhsfCR-1 at 1 µg/mL. Moreover, rhsfCR-1 in the range of 0 to 1 µg/mL also limited the differentiation of miPS-LLCcm cells into vascular endothelial cells probably due to the suppression of self-renewal, which should reduce the number of cells with stemness property. As demonstrated by a soluble form of exogenous Cripto-1 in this study, the efficient blockade would be an attractive way to study Cripto-1 dependent cancer stem cell properties for therapeutic application.

## 1. Introduction

Human cripto-1 (CR-1) or teratocarcinoma-derived growth factor-1 (TDGF-1) is a member of the EGF-CFC protein family, which also includes mouse cripto-1 (Cr-1) [1] and cryptic [2], chicken Cripto [3], Xenopus FRL-1 [4] and zebrafish one-eyed pinhead (oep) [5]. Cripto-1 was first identified and isolated as a cDNA in undifferentiated human and mouse teratocarcinoma cells [6]. Cripto-1 is a multifunctional modulator in early embryonic development where, it is required for correct orientation of the anterior-posterior axis in the mouse embryo [7,8,9]. This oncofetal protein has been emerged as a potential biomarker and expression of this protein has been observed in different types of cancer populations, such as breast carcinomas, colorectal tumors, gastrointestinal carcinomas and teratocarcinomas [10,11,12,13]. Moreover, there is a significantly higher level of expression of CR-1 found in several different types of human malignancies, including ovarian carcinomas, lung carcinomas, pancreatic ductal adenocarcinomas, basal cell carcinomas and bladder carcinomas [14,15,16,17,18].

Human CR-1 is 188 amino acids long, which contains an NH_2_-terminal signal peptide followed by an EGF-like region with a conserved cysteine-rich domain (CFC motif), and its hydrophobic COOH-terminus with a motif of an additional sequence for GPI-anchorage to the cell membrane in lipid rafts [19]. CR-1 induces cellular signaling through several different signaling pathways. One pathway of which is the canonical pathway is the interaction of Cripto-1 with Nodal as a coreceptor that then bind to the receptor complex of ALK4/ALK7 and ActRIIB to stimulate Smad2 phosphorylation and activation of the Smad pathway [20,21,22]. Interestingly, only the presence of EGF-CFC coreceptors is necessary to bind to ALK4/ActRIIB receptor complex for TGF-beta ligands Xenopus Vg1 and its ortholog in mouse GDF1 [23].

Shedding of CR-1 by GPI-phospholipase D [24] can release a soluble form of CR-1 in human milk, serum or in various types of cancer cells [25,26] and is also in the conditioned medium from several cell lines and from cells that are ectopically overexpressing this protein [27]. Moreover, CR-1 can function both as an autocrine (coreceptor in cis) and in trans as (ligand) in a paracrine fashion [28]. Glypican-1/c-Src/MAPK/AKT signaling pathway is another very important mechanism by which CR-1 can signal in a Smad and Nodal independent manner. CR-1 also has the ability to activate the ras/raf/MAPK and PI3-K/AKT/GSK-3β signaling pathways [29,30,31], which also be achieved with a soluble GPI-truncated CR-1 recombinant protein [32]. Activation of c-Src by Cripto-1 leads to activation of ERK1/2 MAPK pathway and PI3K/Akt pathway [33]. GRP78, which is a heat shock protein that is expressed on tumor cells, can amplify both the Nodal/Smad and Smad-independent pathways by binding to CR-1 [34]. Without binding to EGF receptors, Cripto-1 can also induce phosphorylation of Erk-1/2 by Src-dependent phosphorylation of ErbB4 [35].

Recently, we have developed a model of mouse cancer stem cells (CSCs) that are derived from mouse iPSCs in the presence of conditioned medium of Lewis Lung Carcinoma (LLC) cells [36]. miPS-LLCcm cells have been shown to express Nodal and Cr-1 signaling genes implying a possible function of these proteins in the maintenance of stemness in these miPS-LLCcm cells. In this study, we tried to assess the biological significance of soluble exogenous CR-1 in miPS-LLCcm cells.

## 2. Results

### 2.1. Expression of Cr-1 and Related Molecules in miPS-LLCcm Cells

**Figure 1:**
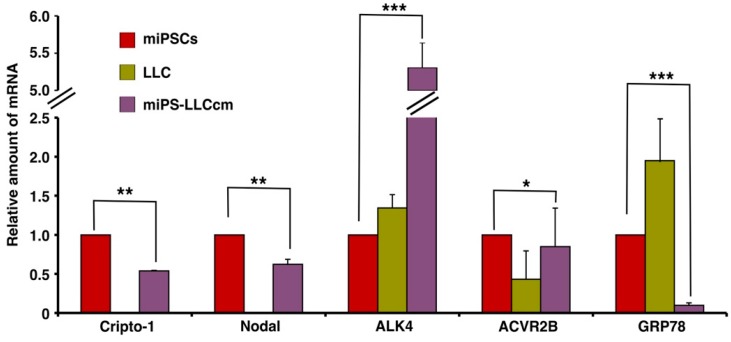
Expression of mRNA for Cr-1 and related molecules in miPSCs, Lewis Lung Carcinoma (LLC) and miPS-LLCcm cells. rt-qPCR was used to assess the relative expression of Cripto-1, Nodal, ACVR2B, ALK4 and GRP78 in these three cell lines. GAPDH was used as an endogenous control and each vertical bar represents the mean ± SD of three data points. The difference between the relative expression in miPS cells and miPS-LLCcm cells is statistically significant as evaluated by Student *t*-test (* *p* < 0.05, ** *p* < 0.01, *** *p* < 0.001).

Mouse miPS-LLCcm cells were assessed for the expression of Cr-1 and Cr-1 signaling proteins, such as Nodal, ACVR2B, ALK4 and GRP78 (Figure 1). The type I ALK4 and type II Activin R2B (ACVR2B), Cripto-1 and Nodal were found to be expressed in the miPSC and miPS-LLCcm cells, but GRP78 was significantly (*p* < 0.001) reduced in the miPS-LLCcm cells than in the LLC cells. In contrast, ALK4 expression was dramatically enhanced in the miPS-LLCcm cells. The Nodal/Cripto-1 signaling through ALK4/Smad2 pathway should be responsible to functionally maintain the self-renewal, proliferation and differentiation of miPS-LLCcm cells. Simultaneously, the expression of Wnt11 and Glypican-1 (Gpc1) were assessed by rt-qPCR (Figure S1). Wnt11 expression was apparently up-regulated in miPS-LLCcm cells while Gpc1 expression was significantly (*p* < 0.01) down-regulated.

### 2.2. rhsfCR-1 Suppressed Differentiation, Proliferation and Sphere Formation of miPS-LLCcm Cells

**Figure 2:**
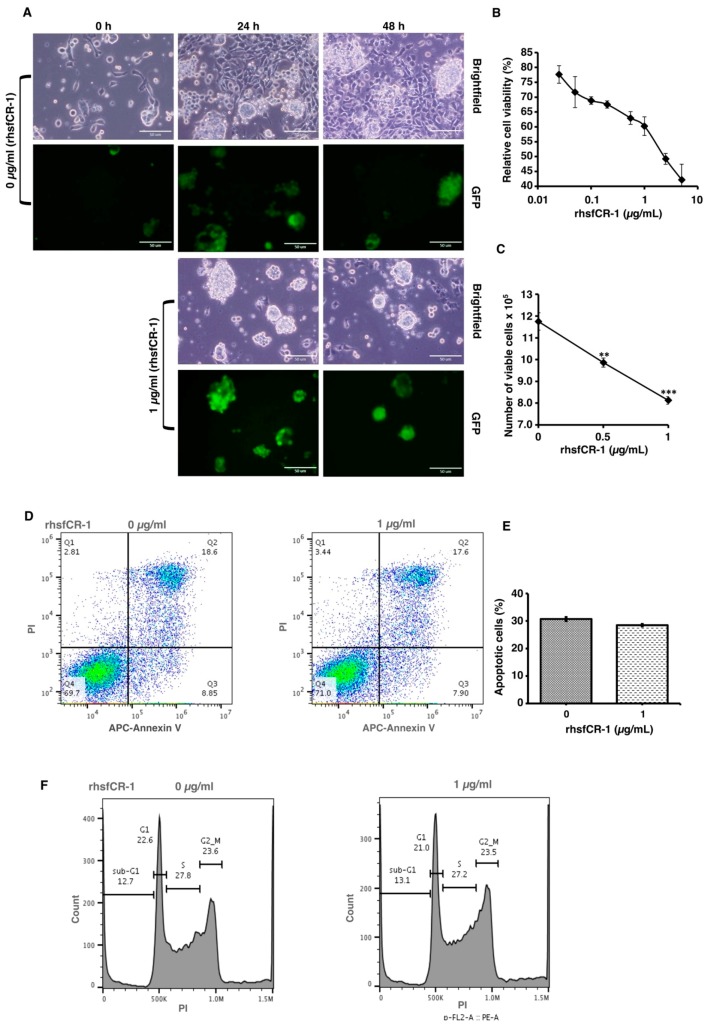

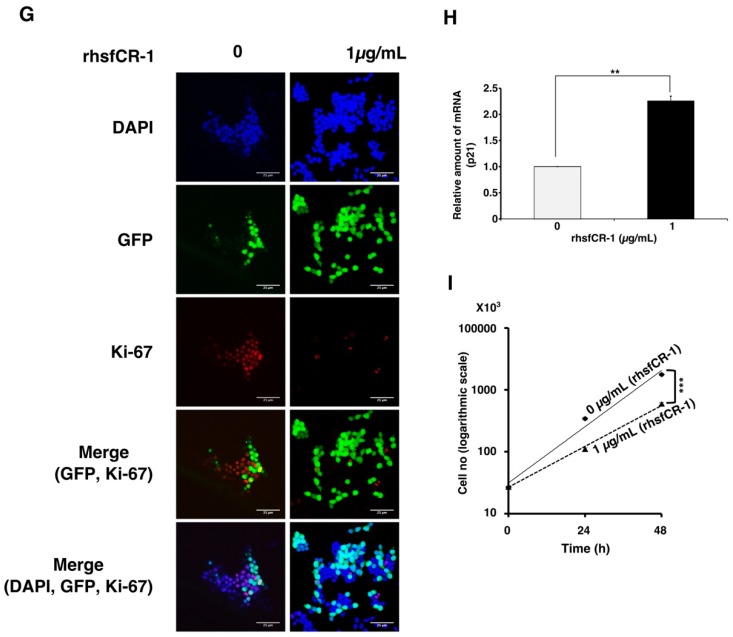
Evaluation of the suppression of growth in miPS-LLCcm cells by rhsfCR-1. (**A**) miPS-LLCcm cells were treated for 48 h with/without rhsfCR-1 (1 µg/mL) and photographed under adherent condition; (**B**) miPS-LLCcm viability were assessed by MTT assay after 24 h treatment with different concentrations of rhsfCR-1. Experiments were repeated three times and the data were plotted as the mean ± SD (*n* = 3); (**C**) live cells were counted after 24 h treatment with either 0.5 µg/mL and 1 µg/mL rhsfCR-1; (**D**) rhsfCR-1 did not induce apoptosis of miPS-LLCcm cells. Apoptosis was assessed after 24 h of treatment with rhsfCR-1 by flow cytometry with double staining with PI and APC-Annexin-V; (**E**) no significant changes in the number of apoptotic cells were found between the treatments with/without rhsfCR-1. Bar plots represent the percentage of apoptotic cells in (**D**); (**F**) rhsfCR-1 did not affect on the cell cycle of miPS-LLC cells. Cells treated with/without rhsfCR-1 were stained with PI and analyzed by flow cytometry; (**G**) confocal observation of immunostaining of miPS-LLCcm cells with anti-Ki-67 antibody together with GFP and DAPI staining; (**H**) rt-qPCR analysis of p21 expression in miPS-LLCcm cells treated with/without rhsfCR-1; (**I**) rhsfCR-1 suppressed the growth of the miPS-LLCcm cells during the time course up to 48 h. Live cells were counted after 24 and 48 h treatments with/without rhsfCR-1. Each bar represents mean ± SD from three independent plates. One-way ANOVA with pairwise multiple comparisons (**C**), Student’s *t*-test (**H**) and Two-way ANOVA (**I**) were used to analyze the level of significance (** *p* < 0.01, *** *p* < 0.001).

**Figure 3:**
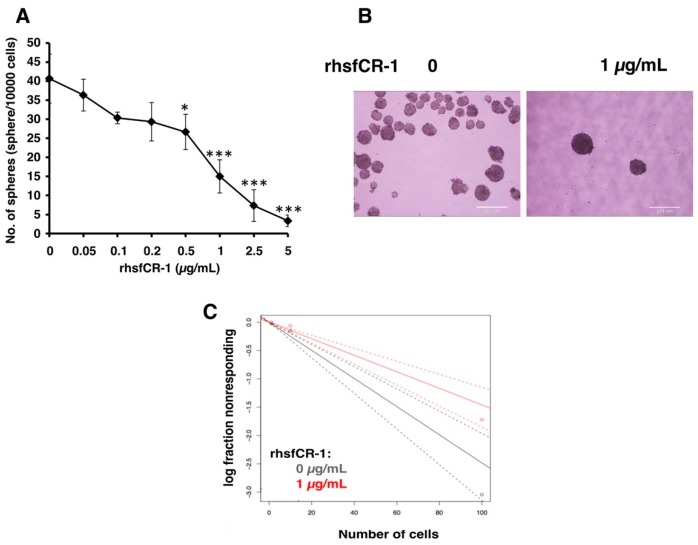
rhsfCR-1 attenuated the sphere formation ability of miPS-LLCcm cells. (**A**) The number of spheres were counted after the treatment with various concentrations of rhsfCR-1 for 1 week under non-adherent condition. Student *t*-test was conducted to analyze the significance (* *p* < 0.05, *** *p* < 0.001); (**B**) spheres in non-adherent cultures in serum free miPS medium supplemented with ITS-x. Spheres were photographed after the treatment with/without rhsfCR-1 (1 µg/mL) for 1 week using sphere formation assay; (**C**) extreme limiting dilution assay assessment of the limiting dilution sphere forming potential showed significantly reduced sphere formation of miPS-LLCcm cells in the presence of rhsfCR-1 at a high cell density per well in 96-well low attachment plates. (See Materials and Methods, Section 4).

To evaluate the function of CR-1 in miPS-LLCcm cells, we designed a soluble form of recombinant human CR-1 protein (rhsfCR-1) (Figure S2) to potentially compete with the binding of endogenous GPI anchored Cr-1 on the cell surface for Nodal complex formation. We analyzed the effects of different concentrations of rhsfCR-1 on the adherent culture of miPS-LLCcm cells. The parental miPSCs used for the conversion into miPS-LLCcm cells [36] carried a GFP reporter gene under the control of Nanog promoter, which turned on the GFP expression in undifferentiated condition, but off in differentiated condition. In the presence of exogenous rhsfCR-1 the miPS-LLCcm cells appeared to be suppressed to undergo differentiations into an adhesive population of cells. Few GFP positive spheres with active Nanog promoter were observed in the presence of rhsfCR-1 (Figure 2A). The proliferation of miPS-LLCcm cells was significantly inhibited by exogenous rhsfCR-1 in a dose-dependent manner in the range of 0 to 5 µg/mL when measured by MTT assay (Figure 2B). The IC_50_ of rhsfCR-1 was estimated approximately 2 µg/mL (125 nM). This inhibitory effect was confirmed by cell counting in the presence of 0.5 and 1 µg/mL of rhsfCR-1 (Figure 2C). Since apoptosis can reduce number of viable cells, we assessed the apoptotic status of miPS-LLCcm cells with/without rhsfCR-1 treatment (Figure 2D). As the results, apoptosis was not induced by rhsfCR-1 (Figure 2E). rhsfCR-1 did not appear to block cell cycle at any particular phase (Figure 2F). The immunoreactivity to the proliferation marker Ki-67 in the cells decreased when treated with rhsfCR-1 (Figure 2G). On the other hand, the expression of p21 was found significantly (*p* < 0.01) up-regulated by 2 folds. (Figure 2H). rhsfCR-1 significantly (*p* < 0.001) slowed the growth during the time course up to 48 h, presumably due to the increased doubling time of the cells (Figure 2I). Further, the effect of exogenous rhsfCR-1 on sphere formation of miPS-LLCcm cells was also evaluated as a CSC property of self-renewal. The number of spheres were significantly down-regulated by rhsfCR-1 in a dose-dependent manner in the range of 0 to 5 µg/mL (Figure 3), which implied that exogenous rhsfCR-1 suppressed the self-renewal potential of miPS-LLCcm cells. The IC_50_ of rhsfCR-1 was estimated to be approximately 0.7 µg/mL (44 nM). Extreme limiting dilution analysis (ELDA) was performed to further evaluate the effects of rhsfCR-1 on stem cell frequency (Figure 3C). Frequency of sphere-forming cells was reduced by rhsfCR-1, dropping from 1/40 to 1/69 (Table S1).

### 2.3. rhsfCR-1 Suppressed Phosphorylation of Smad2 in miPS-LLCcm Cells

**Figure 4:**
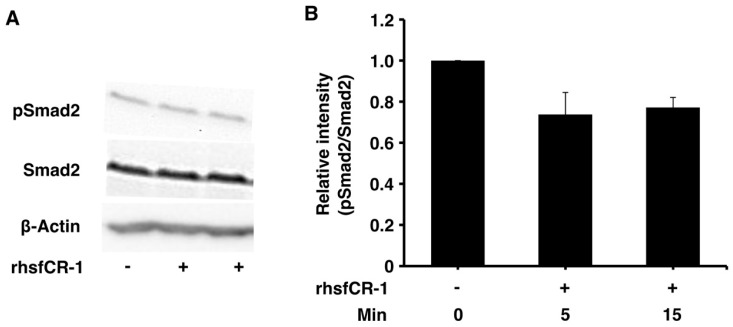
rhsfCR-1 inhibited Smad2 phosphorylation in miPS-LLCcm cells. (**A**) Phosphorylation of Smad2 in miPS-LLCcm cells was assessed by Western Blotting treated with 1 µg/mL rhsfCR-1 for 5 and 15 min in the absence serum. Beta-actin was used as a control. The representative blot was shown; (**B**) the relative intensity of Smad2 phosphorylation bands normalized by each band of Smad2 in Western Blots from three different experiments was densitometrically analyzed using ImageJ software.

Soluble rhsfCR-1 suppressed the phosphorylation of Smad2 in miPS-LLCcm cells implying that the proliferation and sphere formation capacity should be suppressed by the attenuation of ALK4/Smad2 signaling (Figure 4A,B). The phosphorylation of Akt and Erk1/2 was also observed in miPS-LLCcm cells during this experiment (Figure S3). The Nodal/Smad signaling appeared superior to that of Glypican-1/c-Src resulting in the suppression of proliferation.

### 2.4. rhsfCR-1 Suppressed Endothelial Cell Tube Formation by Differentiated miPS-LLCcm

**Figure 5:**
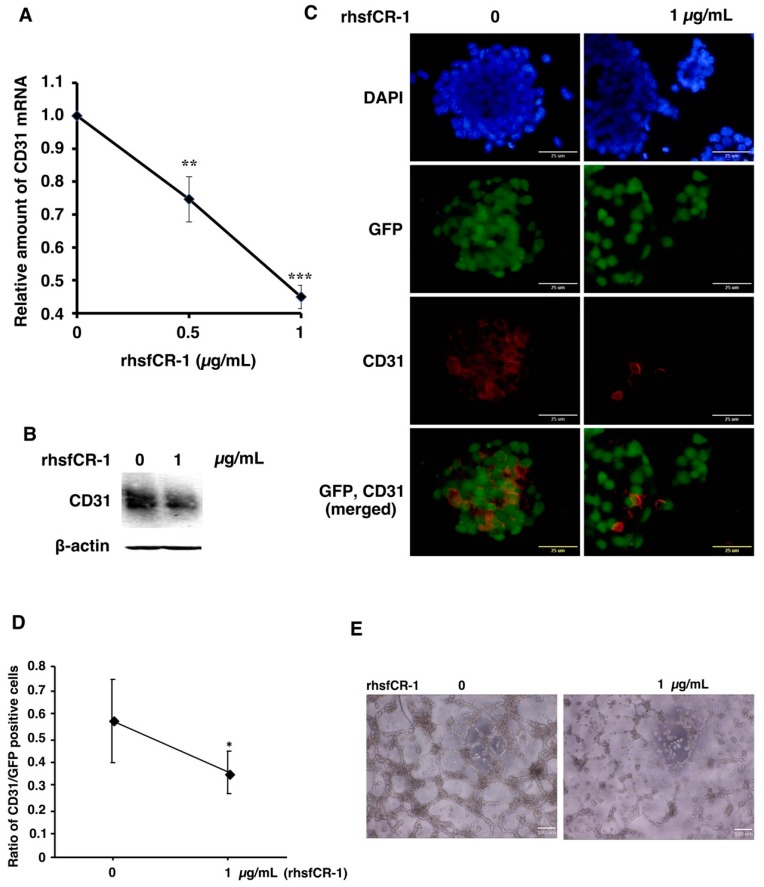
rhsfCR-1 suppressed differentiation into endothelial cells using CD31^+^ phenotype and tube formation by miPS-LLCcm cells. (**A**) Relative expression of CD31 in miPS-LLCcm cells was analyzed by rt-qPCR analysis. The expression level of GAPDH was used as endogenous control. Each plot represents mean ± SD of three data points. One-way ANOVA with pairwise multiple comparison (** *p* < 0.01, *** *p* < 0.001); (**B**) Western blotting analysis showed the reduction of CD31 protein. Beta-actin was used as a control; (**C**) CD31 detected by immunofluorescence (Red) in miPS-LLCcm cells under adherent conditions. CD31 stained with anti-rabbit CD31. The nuclei were counterstained with DAPI (blue); (**D**) ratio of CD31-positive cells over GFP-positive cells in the absence and presence of rhsfCR-1. Student *t*-test was used to analyze the significance (* *p* < 0.05) (**E**) tube formation by miPS-LLCcm cells assessed in the absence or presence of rhsfCR-1 (1 µg/mL).

miPS-LLCcm cells have been reported to differentiate into endothelial cells in the presence of type IV collagen to form tubes in vitro [37,38]. The expression of CD31, which is a marker for endothelial cells, in miPS-LLCcm cells was significantly (*p* < 0.001) down-regulated in a dose-dependent manner by rhsfCR-1 (Figure 5A). The reduction of CD31 was further confirmed by Western Blotting (Figure 5B) and immunocytochemistry with an anti-CD31 antibody (Figure 5C) and by the ratio of CD31-positive cells over GFP-positive cells (Figure 5D). rhsfCR-1 at 1 µg/mL significantly suppressed endothelial cell tube formation by miPS-LLCcm cells in Matrigel (Figure 5E).

### 2.5. rhsfCR-1 Enhanced the Expression of Klf4 and c-Myc in miPS-LLCcm Cells

**Figure 6:**
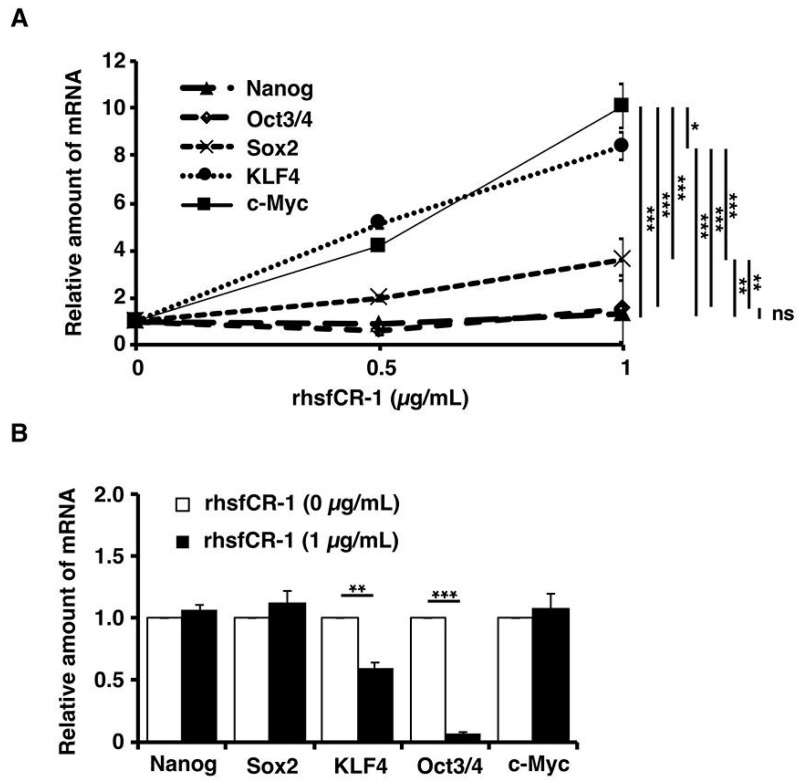
Evaluation of the expression of the stemness markers, Nanog, Oct3/4, Sox2, Klf4 and c-Myc, in miPS-LLCcm cells in adherent culture (**A**) after 24 h of treatment with rhsfCR-1 and in spheres under non-adherent condition (**B**) of miPS-LLCcm cells treated with rhsfCR-1 by rt-qPCR. GAPDH was used as endogenous control and each bar represent mean ± SD of three data points. Two-way ANOVA (**A**) with multiple comparison and Student’s *t*-test (**B**) were conducted to analysis the level of significance (* *p* < 0.05; ** *p* < 0.01; *** *p* < 0.001; ns, not significant).

After miPS-LLCcm cells were treated with different concentrations of rhsfCR-1 in the range of 0 to 1 µg/mL for 24 h under adherent condition, the expression of stemness markers was assessed by rt-qPCR (Figure 6A). The expression of c-Myc and Klf4 genes were found to be up-regulated by 8 to 10 fold, while that of Nanog and Oct3/4 showed little change and that of Sox2 showed mild, but significant (*p* < 0.01), up-regulation when treated with rhsfCR-1. Furthermore, we analyzed the stemness markers in the sphere forming cells of miPS-LLCcm cells under non-adherent condition (Figure 6B). In contrast, significant down-regulation of Oct3/4 (*p* < 0.001) and Klf4 (*p* < 0.01) was observed in the spheres, while no change in the expression of other markers.

### 2.6. rhsfCR-1 Suppressed Migration and Invasion of miPS-LLCcm Cells

**Figure 7:**
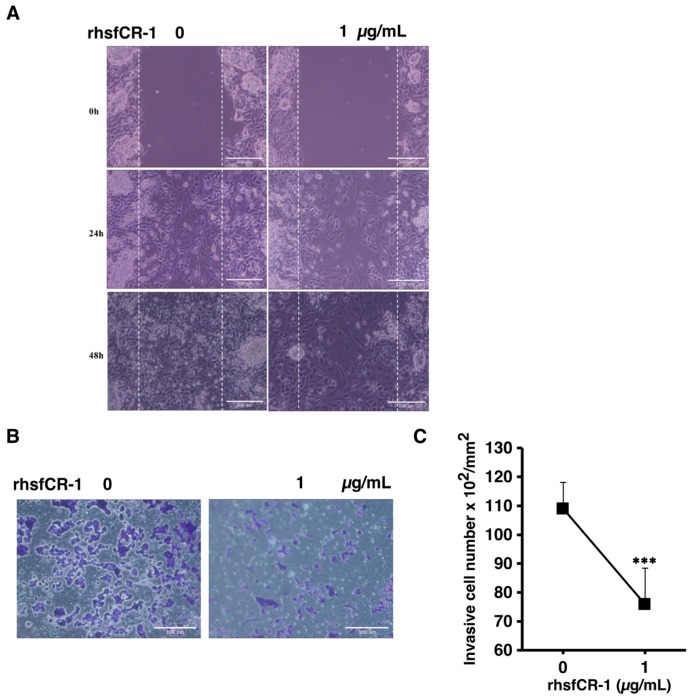
rhsfCR-1 suppressed migration and invasion in miPS-LLCcm cells. (**A**) Wound healing potential was assessed after the treatment without or with rhsfCR-1 (1 µg/mL) for 24 and 48 h; (**B**) invasion ability was assessed with using Matrigel-coated inserts. Invasive cells were stained with Giemsa; (**C**) the graph indicates the results of quantitative analysis of invasive cells stained with Giemsa. The number of stained cells was counted from several different (six) fields. The data were analyzed using two- tailed Student’s *t*-test and are presented as the mean ± SD. *** *p* < 0.001.

In our previous study, we showed that miPS derived CSCs were highly invasive and metastatic [39,40,41]. The migration of miPS-LLCcm cells was significantly affected by rhsfCR-1 at 1 µg/mL in the wound healing assay (Figure 7A). In addition, invasion was also inhibited by 1 µg/mL of rhsfCR-1 (Figure 7B,C).

## 3. Discussion

In this study, we evaluated the effects of a soluble form of human recombinant Cripto-1 (rhsfCR-1) on self-renewal, proliferation, and differentiation potential of CSCs. Previously, full length of human cDNA was expressed in Chinese hamster ovary (CHO) cells and the conditioned medium was used to evaluate the activity on the breast cancer cells [27]. Since the amino-terminal sequence was predicted as a secretion signal, the sequence up to the predicted cleavage site at A^37^ in human CR-1 was removed to design a mature form of hCR-1 when prepared in *Escherichia coli* [42]. CR-1 was found to contain functional omega-sites for GPI anchors at S^169^ and S^161^ [24,43]. We, therefore, designed rhsfCR-1 as a C-terminally truncated form of CR-1 replacing the codon for S^161^ with a stop codon to remove the hydrophobic region of the C-terminal part by site-directed mutagenesis.

The expression of Cripto-1 and Nodal is essential for maintenance of self-renewal and stemness in CSCs [36]. Both are required to enhance sphere formation by activating the Smad2/3 pathway in breast cancer and pancreatic cancer stem cells and are thought to be involved in tumor metastasis [44,45]. Cripto-1 was demonstrated as a functional biomarker for self-renewal property in the CR-1^high^ESCC cells, of which sphere forming activity was significantly decreased when the CR-1 expression was knocked down [46]. By binding to Cripto-1, which is thought to bind to ALK4, but not to ActRIIB, Nodal is able to phosphorylate Smad2/Smad3 signaling pathway through ActRIIB and ALK4/7 receptors and Smad4 forms a complex with these Smad partners [22]. The expression of Cripto-1 and Nodal in miPS-LLCcm cells was observed together with the elevated expression of ALK4 receptor (Figure 1). This observation suggests that the self-renewal potential of miPS-LLCcm cells could feasibly be maintained through a Cripto-1 mediated Nodal/ALK4/Smad2 signaling pathway. Simultaneously, Glypican-1 expression was confirmed in miPS-LLCcm cells implying the presence of Glypican-1/c-Src signaling, which would result in both Erk1/2 and Akt phosphorylation.

When we added high concentrations of exogeneous soluble rhsfCR-1 at the concentration of approximately 0.7 µg/mL (44 nM), we observed 50% inhibitory effect on the self-renewal of miPS-LLCcm cells acting in a dominant negative manner. Since molecular weight of rhsfCR-1 is 16,000, 0.7 µg/mL should be calculated to 2.6 × 10^13^ molecules/mL which should be the number of molecules competing with the membrane-tethered Cr-1. In the assay, approximately 10^4^ cells should be present. Supposing the number of membrane-tethered Cr-1 molecule 10^4^ to 10^5^ on the cell surface, approximately 10^8^ to 10^9^ membrane-tethered Cr-1 should be present. As the result, approximately 2.6 × 10^4^ to 2.6 × 10^5^ folds of molecules appeared to be necessary to exhibit the inhibitory effect. This value is almost 100 to 1000 folds high, when compared with the regular receptor competition with around 10^2^ to 10^3^ folds of molecules. This apparently weak effect might be due to the inefficient interaction of rhsfCR-1 with Nodal/ALK4 and/or the species specificity. From this rough estimation, rhsfCR-1 itself may not be a practically suitable molecule to be developed as therapeutic drug. Probably, smaller size of chemical compounds or monoclonal antibody with high affinity to CR-1 to intercept Nodal/ALK4 binding should be necessary to develop good candidates with 10 to 100 folds lower IC_50_ for the therapeutic purposes.

The suppression of cell proliferation with rhsfCR-1 was not due to the apoptosis or cell cycle arrest at any particular phases, but increased doubling time (Figure 2). Similar results were reported in HeLa cells treated with GST-CR-1 [47]. Moreover, rhsfCR-1 reduced the number of the spheres of miPS-LLCcm cells (Figure 3) in a dose-dependent fashion of rhsfCR-1. Consistently, rhsfCR-1 enhanced the expression of Nodal antagonist Lefty-1 and tumor suppressor Phosphatase and Tensin homolog (PTEN) (Figure S4). Lefty binds ActRIIB and ALK4 and inhibits Nodal signaling whereas increased expression of PTEN reduces activation of Akt inducing anti-proliferative effects. Simultaneously, rhsfCR-1 suppressed the phosphorylation of Smad2 in miPS-LLCcm cells (Figure 4). These results are apparently inconsistent because rhsfCR-1 suppressed Smad2 signaling while activating Ras/Raf/MAPK and PI3K/Akt signaling pathways (Figure S3). Collectively, the self-renewal ability of miPS-LLCcm cells should tightly depend upon the expression of endogenous Cr-1, Nodal and ALK4 resulting in the phosphorylation of Smad2.

In our previous study, the differentiation potential of miPS-LLCcm cells could be demonstrated by spontaneous differentiation miPS-LLCcm cells into CD31 positive endothelial cells that functionally exhibit the ability to show tubes in the presence of type IV collagen [37,38]. In the tumor angiogenesis, Cripto-1 and Nodal are considered to play significant roles [48]. Nodal in breast cancer tissues was also found to be correlated with microvascular density by CD31 staining [49]. High levels of rhsfCR-1 significantly down-regulated the expression of CD31 resulting in the inhibition of differentiation of miPS-LLCcm cells into CD31 positive endothelial cells and subsequent tube formation (Figure 5). Thus, high levels of exogenous soluble rhsfCR-1 was able to attenuate the differentiation of miPS-LLCcm cells into vascular endothelial cells.

CR-1 is thought to play a vital role in the migration and invasion of several different types of cancers, such as esophagus squamous cell carcinoma and prostate carcinoma [46,50]. CR-1 and Nodal are capable of enhancing the progression and invasion of glioma cells by activating Smad signaling pathway [51]. miPS-LLCcm cells are highly invasive and exhibit high metastatic potential in vivo [39,41]. Interestingly, in adherent condition, rhsfCR-1 enhanced the expression of c-Myc and Klf4, which promote epithelial mesenchyme transition (EMT), in a dose-dependent manner while the expression of Nanog Sox-2 and Oct3/4, which should support the stemness of miPS-LLCcm cells, was also affected slightly (Figure 6A). Like other cancer stem cells, miPS-LLCcm cells create their own niche to maintain themselves in the hierarchy of differentiating CSCs. miPS-LLCcm cells differentiate into the heterogenous population, including vascular endothelial cells [37,38] as well as cancer associated fibroblast cells [52] in tumor microenvironment. However, the heterogeneity that should be related with cell plasticity maintaining stem cell phenotype [41,53] should explain the up-regulation of the stem cell markers. In fact, comparing the results between the adhesive culture and non-adhesive culture, we found different patterns in the expression of stemness markers (Figure 6A,B). Especially, Klf4 and Oct3/4 were significantly down-regulated in non-adhesive culture. Although we cannot speculate the phenotypes of each heterogeneous component of plastic cells, these findings should imply the presence of cancer stem cell plasticity lying in the heterogeneity.

In contrast, rhsfCR-1 clearly inhibited the migration and invasion of the miPS-LLCcm cells (Figure 7). Although we assessed the expression of Snail, TWIST1/2, MMP2/9 and E-cadherin, none of these expressions was found correlated with the suppression of the migration (data not shown). Since the involvement of Wnt signaling might be another cause [54], the effect of rhsfCR-1 was assessed on the expression of beta-catenin, which was found significant (*p* < 0.01) in the down-regulation at 1 µg/mL (Figure S5). This down-regulation may result in the suppressing effects of rhsfCR-1 on both self-renewal and proliferation of miPS-LLCcm cells. This suppression could then potentially lead to reduce the number of stem cells that are able to differentiate and promote EMT.

As discussed above, rhsfCR-1 itself is not an ideal form for the development as a pharmaceutical drug, due to the inefficient interaction with Nodal/ALK4. Simultaneously, the amount of rhsfCR-1 for in vivo study was estimated to be gram order comparing with our results of drug delivery of small anti-cancer drug [55]. The estimated amount of rhsfCR-1 protein is quite too huge to prepare and not practically available for i.v. injection per mouse body. In this kind of in vivo study, an efficient antagonist against CR-1, of which affinity is high enough to inhibit CR-1 from binding to Nodal/ALK4 exhibiting IC_50_ 10–100 folds lower than that of rhsfCR-1, should be necessary to demonstrate the efficacy.

## 4. Materials and Methods

### 4.1. Mutagenesis, Expression and Purification of Truncated Soluble rhsfCR-1

*E. coli* BL21(DE3) harboring pLysS and *E. coli* DH5α were cultured in LB media with ampicillin (100 µg/mL) and/or chloramphenicol (10 µg/mL). High pure plasmid isolation kit ver. 09 (Roche Diagnostics GmbH, Mannheim, Germany) was used for plasmid DNA isolation. Plasmid pBO188 [42] carrying a cDNA coding mature form of hCR-1 (151 aa) was used as a template for polymerase chain reaction (PCR) based in vitro site-directed mutagenesis using KOD-Plus-Neo (Toyobo, Osaka, Japan). We have designed the primers (Table S2) to substitute the codon for S^161^ to a stop codon so that the omega site of GPI anchor and the hydrophobic sequence in the C-terminus would be removed. After the digestion with Dpn1 (New England Biolab, Ipswich, MA, USA) treatment, the resultant plasmid pBO1801 was obtained by transforming *E. coli* DH5α.

DNA sequencing was done with ABI prism bigdye terminator V1.1 on ABI prism genetic analyzer 3130 (Applied BioSystems, Foster City, CA, USA). We used 4PeaKs software to analyze the sequenced data. After confirmation, *E. coli* BL21 (DE3)/pLysS were transformed with the plasmid pBO1801. In order to produce rhsfCR-1, the transformant was cultured at 37 °C in LB medium and the recombinant expression was induced with 0.4 mM isopropyl β-D-1-thiogalactopyranoside (IPTG) when the cell density got to 0.6 OD_600_ after shaking (Bioshaker G. BR-300, TAITEC Co., Saitama, Japan). Cells was harvested by 8000 rpm for 20 min at 4 °C after 3 h of induction and stored at −80 °C until use. Harvested cells were suspended in lysis buffer (20 mM Tris-HCl, pH 8.0, 150 mM NaCl, 5% glycerol, 0.1% Triton-x-100, 1 mM PMSF) and incubated at 4 °C for mixing by Rotator RT-5 (TAITEC Co., Saitama, Japan) for 1 h and sonication was done at 50% duty cycle, 4 min on ice with 1 min interval using Misonix Astrason 3000 (Misonix Inc., Farmingdale, NY, USA). The insoluble fraction produced inclusion bodies were then collected by centrifugation at 15,000 rpm with Himac CR20 (Hitachi-Koki, Ibaragi, Japan) for 15 min at 4 °C. The precipitates were suspended and washed in a solution containing 0.5% Triton X-100 followed by brief sonication. After several washes the recombinant protein in the inclusion bodies was solubilized in a buffer containing 8 M urea.

Denatured protein was then passed through Hitrap chelating Hp column with immobilized nickel (GE Healthcare, Chicago, IL, USA) for His-tag purification in 8 M urea. The eluted protein solution was purified again in TOYOPEARL CM650M (Tosoh Co., Tokyo, Japan) by cation exchange chromatography using a Biologic LP (BioRad Labs, Hercules, CA, USA). The adsorbed denatured protein was eluted with a linear gradient of NaCl concentration (0 to 1 M) in sodium acetate buffer (pH 5.0) containing 6 M urea.

### 4.2. Refolding and Dialysis of rhsfCR-1 Protein

The fractions of target protein eluted from TOYOPEARL CM650M column were pooled and pH of the pooled fractions was adjusted to pH 8.5 with Tris-HCl pH 8.5. Then the protein solution was degassed and beta-mercaptoethanol was added to a final concentration of 0.1 M to reduce rhsfCR-1 with an incubation at 40 °C for 1 h. After that protein solution was refolded by 100 mM Tris-HC1 (pH 8.0) containing 0.5 mM oxidized glutathione (GSSG) at 4 °C. After 16 h incubation refolded protein was collected by centrifugation at 12,000 rpm for 15 min at 4 °C. Dialysis with SeamLess cellulose tubing size 24/32 (Viskase Co. Inc., Lombard, IL, USA) was conducted against 1xPBS and with three changes of buffer with stirring at 4 °C overnight. After removal of insoluble materials, the protein solution was sterilized through a 0.2-micron filter.

### 4.3. SDS-PAGE and Estimation of Protein

Protein purification was monitored by SDS-PAGE (15% gel). The gel was stained with Coomassie brilliant blue R250 (CBB, Nacalai Tesque Inc., Kyoto, Japan). For protein quantification, we used Bio-Rad protein assay dye reagent concentrate with bovine IgG (BioRad Labs, Hercules, CA, USA) as a standard.

### 4.4. Cell Culture

Mouse LLC cells were obtained from the American Type Culture Collection (ATCC, Manassas, VA, USA) and were maintained in Dulbecco’s Modified Eagle’s Medium-high glucose (DMEM, Sigma-Aldrich, St. Louis, MO, USA) containing 10% FBS (Thermo Fisher Scientific, Waltham, MA, USA) and 100 U/mL penicillin/streptomycin (Wako, Osaka, Japan). miPS cells (iPS MEF-Ng-20D-17) (Riken Cell Bank, Ibaraki, Japan) [56] were cultured in miPS media (DMEM containing 15% FBS, 0.1 mM MEM Non-Essential amino acids, (NEAA, Thermo Fisher Scientific, Waltham, MA, USA), 2 mM L-Glutamine (Nacalai Tesque, Kyoto, Japan), 0.1 mM 2-mercaptoethanol (Sigma-Aldrich, St. Louis, MO, USA), 50 U/mL penicillin/ streptomycin (Wako, Osaka, Japan) and 1000 U/mL of Leukemia inhibitory factor (LIF, Merck Millipore, Burlington, MA, USA)) on a feeder layer of mitomycin-treated mouse embryonic fibroblast (MEF) cells (REPROCELL Inc., Kanagawa, Japan). miPS-LLCcm cells have been established as a mouse cancer stem cell model by converted from miPS cells [36,37,38,39,41]. The capacity of self-renewal was confirmed by sphere forming potential in non-adhesive conditions. Differentiation potential was confirmed by the morphological change into the epithelial-like flat cell shape in adhesive conditions and into CD31 positive vascular endothelial cells forming tubes in Matrigel, as well as in vivo. miPS-LLCcm cells are highly tumorigenic when subcutaneously transplanted into Balb/c nude mice. In addition, miPS-LLCcm cells express the marker genes, such as Nanog, Rex1, Eras, Esg1, Cripto-1, CD44 and ALDH1, which are considered associated with cancer stem cell properties. miPS-LLCcm cells were cultured in miPS media without LIF. All cells were maintained at 37 °C in the atmosphere of 5% CO_2_. Medium was changed every two days with fresh media if necessary.

### 4.5. Cell Proliferation and Viability Assays

To measure viability of cells at different experimental conditions, cells were seeded in 96-well plates (5000 cells/well) and treated as per the requirement. After 24 h, cell viability was assayed calorimetrically with Thiazolyl Blue Tetrazolium Blue (MTT, Sigma-Aldrich, St. Louis, MO, USA) [57] at 570 nm and cell viability was calculated relative to the untreated cells. Cell proliferation was also measured by counting live cells under light microscope using trypan blue dye exclusion method.

### 4.6. Apoptosis and Cell Cycle Analysis

Level of apoptosis was estimated by flow cytometry using APC Annexin V Apoptosis Detection Kit with propidium iodide (PI) (BioLegend, San Diego, CA, USA) as per the manufacturer’s protocol. Briefly, cells were seeded in 6-cm dishes and after 24 h of rhsfCR-1 treatment, total cells were harvested, washed, stained with APC-Annexin V and PI and analyzed by BD Accuri^TM^ C6 plus flow cytometer (Becton & Dickinson, Franklin Lakes, NJ, USA). Data of each experiment was analyzed using FlowJo^®^ software (FlowJo, LLC, Ashland, OR, USA). Cells stained with PI was used for cell cycle analysis. miPS-LLCcm cells were seeded in 6-cm dish and after 24 h of treatment, cells were harvested, fixed in cold 70% ethanol and after RNase (Nippon Gene, Tokyo, Japan) treatment, cells were stained with PI and analyzed by flow cytometry and cell cycle analysis was performed using FlowJo^®^ Software (ver. 10.4.2, FlowJo, LLC, Ashland, OR, USA).

### 4.7. Sphere Formation and Extreme Limiting Dilution Analysis

miPS-LLCcm cells (4 × 10^4^) were seeded on 6-cm ultra-low attachment dishes (Corning Inc., Corning, NY, USA) or on 24 well ultra-low attachment dishes (Corning Inc., Corning, NY, USA) (1 × 10^4^ cells/well) in FBS-free DMEM supplemented with Insulin-Transferrin-Selenium-X (ITS-X; 1/100 *v*/*v*) (Life Technologies, Carlsbad, CA, USA), 0.1 mM NEAA, 2 mM L-glutamine 50 U/mL penicillin/streptomycin (Wako, Osaka, Japan) and 0.1 mM 2-mercaptoethanol (Sigma-Aldrich, St. Louis, MO, USA). After 5–7 days, the number of spheres was counted, and images were acquired using CKX41 inverted microscope (Olympus, Tokyo, Japan) or an IX81 inverted microscope (Olympus, Tokyo, Japan) equipped with a light fluorescence device (Olympus, Tokyo, Japan). For limiting dilution analysis, we seeded 100, 10 and 1 cells in 96-well low attachment plates (EZ-BindShut^TM^SP, Asahi Glass Co., Ltd., Tokyo, Japan) in the above medium and after 1 week of incubation stem cell frequency was calculated with software available at http://bioinf.wehi.edu.au/software/elda/index.html [58].

### 4.8. In Vitro Tube Formation Assay

For in vitro tube formation assay, 96-well plates were coated with growth factor reduced Matrigel (Corning Inc., Corning, NY, USA) and 5 × 10^4^ cells were seeded in 50 µL of Endothelial Cell Growth Medium 2 (PromoCell, Heidelberg, Germany) for 24 h with growth supplements: Human Epidermal Growth Factor (hEGF; 5 ng/mL), Vascular Endothelial Growth Factor (VEGF; 0.5 ng/mL), R3-Insulin-like Growth Factor-1 (R3-IGF-1; 20 ng/mL), Ascorbic Acid (1 µg/mL), Hydrocortisone (0.2 µg/mL), human basic Fibroblast Growth Factor (hbFGF; 10 ng/mL), Heparin (22.5 µg/mL), FBS (0.02 mL/mL), Gentamicin/Amphotericin-B (GA) and pictures were taken by Olympus IX81 microscope (Olympus, Tokyo, Japan).

### 4.9. Immunofluorescence Staining

The cells were seeded at a density of 1 × 10^5^ cells on gelatin coated cover glasses, after incubation, cells were washed with 1xPBS and fixed with 4% paraformaldehyde for 20 min at room temperature, and subsequently permeabilized using 0.05% Triton-X 100 (Nacalai Tesque, Kyoto, Japan) in PBS for 5 min. The cells were incubated in blocking solution (PBS supplemented with 10% FBS) for 1 h. The cells were incubated overnight at 4 °C with rabbit polyclonal anti-CD31 antibody (1:100, ab28364, Abcam, London, UK) and after washing again incubated with Alexa Fluor 555 conjugated anti-rabbit IgG (1:500, A21428, Life technologies, CA) for 1 h. After removal and proper washing of secondary antibody, nuclei were counterstained with 4′,6-diamino-3-phenylidole, dihydrochloride (DAPI) (Sigma-Aldrich, St. Louis, MO, USA). The cells were mounted on glass sides using Vectashield mounting medium (Vector Labs, Burlingame, CA, USA) and images were taken by Olympus IX81 microscope. For the immunostaining for Ki-67, cells were incubated with rabbit polyclonal anti-Ki67 antibody (1:500, ab66155, Abcam, London, UK) and same (as for CD31) secondary antibody was used and fluorescent signal was imaged using a laser scanning confocal microscope (FV-1000, Olympus, Tokyo, Japan).

### 4.10. RNA Extraction and Quantitative Reverse Transcription PCR (rt-qPCR)

RNAeasy Mini kit (QIAGEN, Hilden, Germany) was used to isolate total RNA from cells and the extracted RNA was treated with DNase I (Promega, Fitchburg, WI, USA). One μg of RNA was reverse transcribed using GoScript™ Reverse Transcription System (Promega, Fitchburg, WI, USA). qPCR assays were done by LightCycler 480 II (Roche Diagnostics GmbH, Mannheim, Germany) using LightCycler 480 SYBR green I Master Mix (Roche Diagnostics GmbH, Mannheim, Germany) according to the manufacturer’s instructions. Gene expression level was normalized with that of Glyceraldehyde-3-phosphate dehydrogenase GAPDH mRNA. The primers used for the rt-qPCR analysis are listed in Supplementary Table S2.

### 4.11. In Vitro Migration and Invasion Assays

Scratch wound assay (migration) was carried out to observe of cell migration. miPS-LLCcm cells were cultured in miPS medium containing 15% FBS until monolayer cells reached 70–80% confluence. Then culture medium was replaced with miPS medium containing 5% FBS. Wounds were created by scratch with 200 microliter-pipette tips. Wound healing processes were photographed at 0, 24 and 48 h under Olympus IX80 inverted microscope. Cell invasion potential of miPS-LLCcm was assessed using Falcon cell culture inserts (Corning Inc., Corning, NY, USA). First, the chamber was prepared by coating with ice-cold Matrigel. Then, 5 × 10^4^ cells were seeded onto the coated Matrigel in upper chamber with 500 μL of DMEM, and miPS medium supplemented with 15% FBS was added to the lower chamber. Untreated and rhsfCR-1 treated cells were allowed to grow for 72 h. Non-invasive cells were removed by wiping the upper surface and invaded cells on the lower surface were fixed in 4% paraformaldehyde (Nacalai Tesque, Kyoto, Japan) for 5 min and in methanol (Wako, Osaka, Japan) for 20 min respectively. The cells were stained with Azure EMB Giemsa (Merck Millipore, Burlington, MA, USA).

### 4.12. Western Blotting

For Smad2, Akt and Erk1/2 phosphorylation assay, miPS-LLCcm cells were grown in miPS medium supplemented with 15% FBS until they became confluent. After the cells were starved without FBS for 6 h and washed with PBS and then stimulated with rhsfCR-1 for 5 and 15 min in the medium without FBS. Proteins following the SDS-PAGE were transferred to polyvinylidene difluoride (PVDF) membranes (Merck Millipore, Burlington, MA, USA) and probed with antibodies against anti-Akt (1:1000, 4691S), anti-p-Akt (S473) (1:2000, 4060S), anti-Smad2 (1:1000, 5359S), anti-p-Smad2 (S465/467) (1:1000, 3108S), anti-p44/42 MAPK (Erk1/2) (1:1000, 4695S), anti-P-p44/42 MAPK (1:2000, 4370S) (Cell Signaling Technology, Inc., Beverly, MA, USA); anti-CD31 (1:500, ab28364, Abcam, UK) followed by horseradish peroxidase conjugated anti-rabbit IgG (1: 2000-1: 5000, 7074, Cell signaling Technology, Inc., Beverly, MA, USA). ImageJ software was used to densitometrically analyze the intensity of western blot bands and beta-actin (1:1000, 4970S, Cell Signaling Technology, Inc., Beverly, MA, USA) was used as a normalization control.

### 4.13. Statistical Analysis

Quantitative data were collected from independent experiments performed at least three times and expressed as mean ± SD and statistically analyzed by Student’s *t*-test, as well as one-way and two-way ANOVA with multiple comparisons (* *p* < 0.05, ** *p* < 0.01 and *** *p* < 0.001).

## 5. Conclusions

It is believed that the self-renewal and differentiation properties of CSCs are regulated and maintained by the CSC niche, which should include various signaling pathways related with Cripto-1, such as Glypican-1/Src/Akt and Wnt/Frizzled/beta-catenin, as well as Nodal/ALK4/Smad2. Here, in this paper we have successfully demonstrated competitive exogenous soluble form of CR-1 suppress the self-renewal of CSCs. Cripto-1 appears to contribute to self-renewal in CSCs through Nodal/ALK4/Smad2 signaling pathway. Although rhsfCR-1 successfully demonstrated the blockade of interaction between Cripto-1 and Nodal/ALK4, which should be effective to suppress the self-renewal of CSCs, rhsfCR-1 itself is not a practical molecule for therapy, due to the inefficient interaction with Nodal/ALK4. The design of efficient targeting of CR-1 by small chemical compounds or monoclonal antibodies exhibiting the IC_50_ ranging in picomol order will be a promising candidate molecule to treat CSCs suppressing self-renewal, proliferation, cellular motility and EMT.

## Supplementary Materials

Supplementary materials can be found at http://www.mdpi.com/1422-0067/19/11/3345/s1.
