# Supplementary material for: Exogenous Cripto-1 Suppresses Self-Renewal of Cancer Stem Cell Model
Source: Int J Mol Sci. 2018 Oct 26;19(11):3345. doi: 10.3390/ijms19113345 (PMC6274844; doi:10.3390/ijms19113345)
Supplement: Supplementary file 1 [file ijms-19-03345-s001.pdf]

International Journal of *Molecular Sciences*

MDPI

Supplementary Materials: Exogenous Cripto-1 Suppresses Self-renewal of Cancer Stem Cells Model

Md Jahangir Alam, Ryota Takahashi, Said M Afify, Aung Ko Ko Oo, Kazuki Kumon, Hend M Nawara, Aprilliana Cahya Khayrani, Juan Du, Maram H Zahra, Akimasa Seno, David S. Salomon and Masaharu Seno

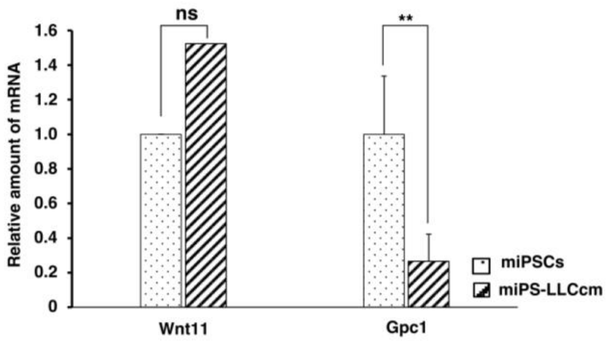

Figure S1. Evaluation of the expression of Wnt11 (**A**) and Gpc1 (**B**) in miPS-LLCcm cells by rt-qPCR. GAPDH was used as endogenous control and each bar represent mean ± SD of three data points. Student's t-test was used to measure the level of significance. (**p < 0.01; ns, not significant).

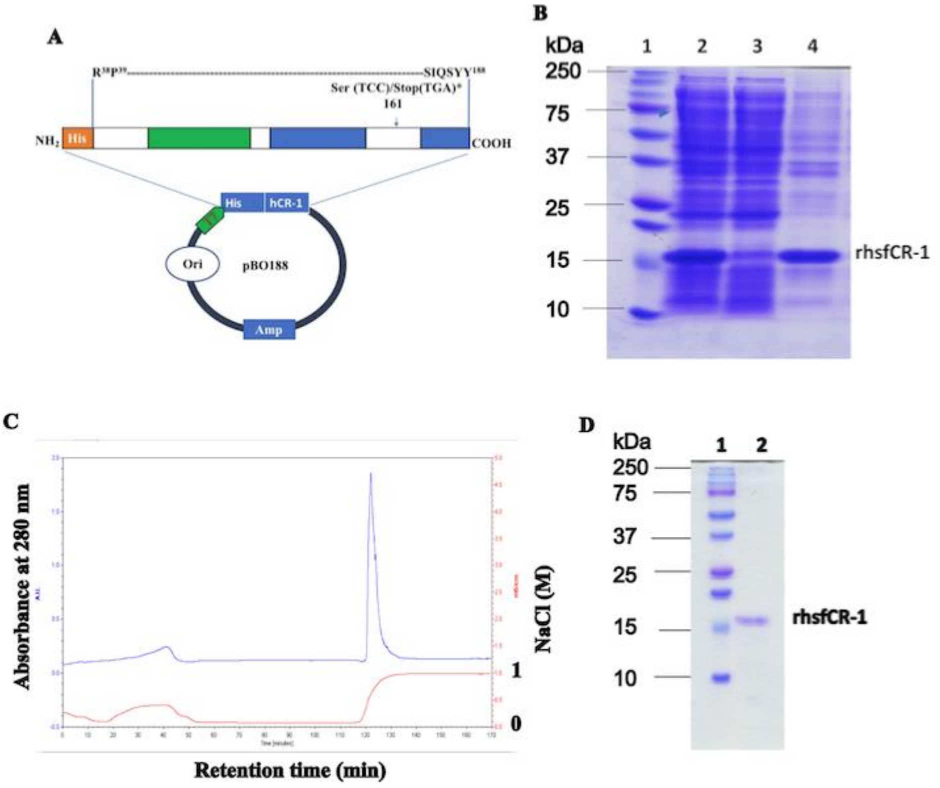

Figure S2. Expression and purification of rhsfCR-1. (**A**) Schematic drawing of recombinant human Cripto-1 construct fused to a His-tag. Site-directed mutagenesis was carried out in plasmid pBO188 to have S[161] codon to a stop codon at 161 to express under the control of T7 promoter (*resultant plasmid named as pBO1801); (**B**) SDS-PAGE analysis of the transformant expressing rhsfCR-1. Lane 1, molecular weight markers; 2, whole cell lysate; 3, soluble fraction of whole cell lysate; 4, inclusion bodies; (**C**) chromatographic isolation of rhsfCR-l through CM650M cation exchange column under a

gradient of NaCl; (**D**) SDS-PAGE of purified refolded rhsfCR-1 protein. Lane 1, molecular weight markers; 2, purified rhsfCR-1.

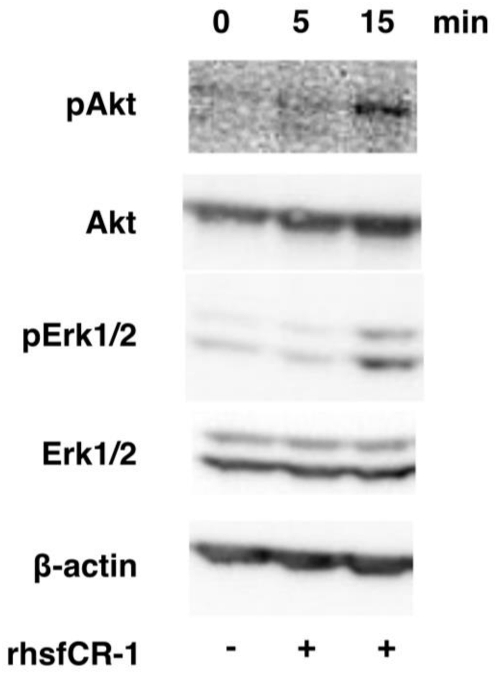

Figure S3. Akt and Erk1/2 phosphorylation assay in miPS-LLCcm cells. Western blot was performed to detect phosphorylation status of Akt and Erk1/2 in miPS-LLCcm cells, treated with 1 μg/ml rhCR-1 for 5 and 15 min in the absence of serum after serum starvation. Beta-actin was used as a control.

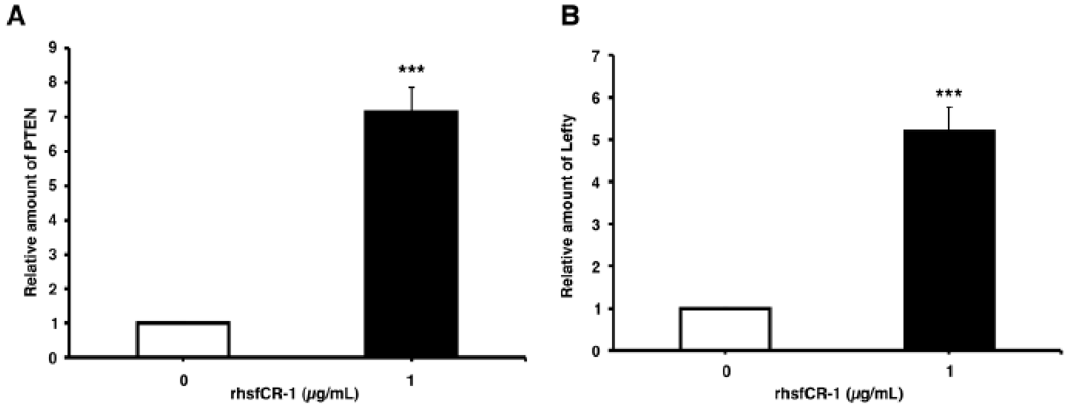

Figure S4. Evaluation of the expression of PTEN (**A**) and Lefty (**B**) in miPS-LLCcm cells on adherent culture after 24 h of treatment with rhsfCR-1 by rt-qPCR. GAPDH was used as endogenous control and each bar represent mean ± SD of three data points. Student t-test were conducted to analysis the level of significance (*** $p < 0.001$).

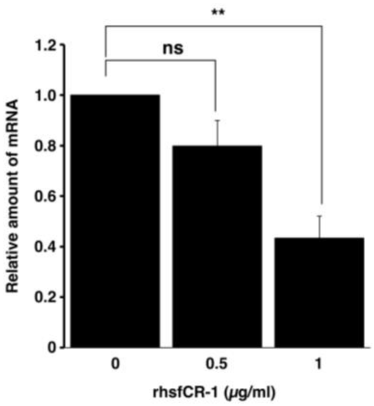

Figure S5. rt-qPCR analysis of Beta-catenin in miPS-LLCcm cells on adherent culture after 24 h of treatment with rhsfCR-1. GAPDH was used as endogenous control and each bar represent mean ± SD of three data points. Student's t-test was used to measure the level of significance. (**$p<0.01$; ns, not significant).

Table S1. Limiting dilution analysis for sphere formation

Group	Cells plated per well	Number of wells positive	Sphere-forming frequency (95% CI)
0 μg/ml	100	80/84	
0 μg/ml	10	12/84	1/40 (1/51 - 1/32)
0 μg/ml	1	2/84	
1 μg/ml	100	69/84	
1 μg/ml	10	5/84	1/69 (1/87 - 1/54) **
1 μg/ml	1	0/84	

Table S2. List of primers used in the experiments.

No	Names	Forward Primer Sequence (5'-->3')	Reverse Primer Sequence (5'-->3')
1	Nanog	AGGGTCTGCTACTGAGATGCTCTG	AACCCAAGCACGTATCAGGG
2	Oct3/4	TCTTTCCACCAGGCCCCCGGCTC	TGCGGGCGGACATGGGGAGATCC
3	Sox2	TAGAGCTAGACTCCGGGCGATGA	TTGCCTTAAACAAGACCACGAAA
4	Klf4	GGACTTACAAAATGCCAAGGGGTG	TCGCTTCCTCTTCCTCCGACACA
5	c-Myc	TGACCTAACTCGAGGAGGAGCTGG AATC	AAGTTTGAGGCAGTTAAAATTATGGCTGA AGC
6	E-cadherin	AACCCAAGCACGTATCAGGG	GGGGTCTGTGACAACAACGA
7	Snail	GGAGTTGACTACCGACCTTGC	TGGAAGGTGAACTCCACACAC
8	MMP2	CAAGTTCCCCGGCGATGTC	TTCTGGTCAAGGTCACCTGTC
9	MMP9	CTGGACAGCCAGACACTAAAG	CTCGCGGCAAGTCTTCAGAG
10	Twist1	GCCGGAGACCTAGATGTCATTGT	TTAAAAGTGTGCCCCACGCC
11	Twist2	CTCACGAGCGTCTCAGCTAC	TTGTCCAGGTGCCGAAAGTC
12	CD31	AAC TCC TTC ACC ATC AAC AGC ATC	AAT GAC GTA GCT CTC GGT GTG
13	Cripto-1	GGGGTACTTCTCATCCAGTGTG	ACTGTTCTGTATCCCCACGG
14	Nodal	ATTTGCCAGACAGAAGCCAAC	TCCTCCACAATCATGTCCTTG
15	ALK4	CTCAATGGAAGGGTCGGAGG	GGGACCAAACGATACATGGC
16	ACVR2B	ACGACTTTGTGGCTGTGAAG	CAGTTCGTTCCACGTGATGATG
17	GRP78	GTGTGTGAGACCAGAACCGT	GCAGTCAGGCAGGAGTCTTA
18	GADPH	AACGGCACAGTCAAGGCCGA	ACCCTTTTGGCTCCACCCTT
19	PTEN	CCTGCAGAAAGACTTGAAGGTGT	CAAAAGGATACTGTGCAACTCTGC
20	Lefty1	AGCTCAAGGCAATTGTGACC	TCATCTCTGAGGCGACACAC
21	Wnt11	ACACTGTAAACAGCTGGAGGG	CGTGTACCTCTCTCCAGGTCAA
22	Gpc1	AGGATGCTAGTGATGACGGC	AGCTGAGGTCTTCTGTCCCT
23	p21	GCCCGAGAACGGTGGAACTT	GACAAGGCCACGTGGTCCTC
24	Beta catenin	TCCCATCCACGCAGTTTGAC	TCCTCATCGTTTAGCAGTTTTGT
25	Cripto-1 (mutagenesis)	GAG CAC CTC GTG GCT TGA AGG ACT CCA GAA CTA C	GTA GTT CTG GAG TCC TTC AAG CCA CGA GGT GCT C
26	T7 (Sequencing)	Promoter primer (TAATACGACTCACTATAGG)	Terminator primer (GCTAGTTATTGCTCAGCGG)

© 2018 by the authors. Submitted for possible open access publication under the terms and conditions of the Creative Commons Attribution (CC BY) license (http://creativecommons.org/licenses/by/4.0/).
